# Predicting Technology Use Among Older Adults During the COVID-19 Pandemic With Community Participation

**DOI:** 10.1093/geroni/igab046.2164

**Published:** 2021-12-17

**Authors:** Brittany Drazich, Laura Samuel, Thomas Cudjoe, Melissa Hladek, Sarah Szanton, Qiwei Li

**Affiliations:** 1 Johns Hopkins University School of Nursing, Baltimore, Maryland, United States; 2 Johns Hopkins University, Baltimore, Maryland, United States; 3 Johns Hopkins University School of Medicine, Baltimore, Maryland, United States; 4 Johns Hopkins School of Nursing, Baltimore, Maryland, United States

## Abstract

Technology use is important for older adults, particularly in the pandemic. The pattern of technology use among older adults varies significantly. We hypothesized that limitations of activities of daily living (ADL), wellbeing, and community participation of community-dwelling older adults before the pandemic would predict technology use during the pandemic. National Health and Aging Trends Study data on 2924 older adults were utilized. Adjusted for age, gender, race, education, marital status, and chronic conditions, previous well-being predicted more online social activities (OR=1.03, p =.03); previous ADL limitations predicted more telehealth use (OR=1.11, p=.014); and previous community participation predicted: learning new technologies (OR=1.46, p <.001), more telecommunication (OR=1.12, p=.007), more online social activity (OR=1.58, p<.001), and more telehealth use (OR=1.09, p= .04). The results of this study imply that high community participation promotes older adults’ transition to technology use. Older adults with low participation may need extra attention for such a transition.

